# (*S*)-Methyl 2-benzamido-3-(3,4-dimeth­oxy­phen­yl)propano­ate

**DOI:** 10.1107/S1600536811052767

**Published:** 2011-12-21

**Authors:** Tricia Naicker, Thavendran Govender, Hendrick. G. Kruger, Glenn E. M. Maguire

**Affiliations:** a School of Pharmacy and Pharmacology, University of KwaZulu-Natal, Durban 4000, South Africa; b School of Chemistry, University of KwaZulu-Natal, Durban 4000, South Africa

## Abstract

The dimethoxypbenzene ring in the title compound, C_19_H_21_NO_5_, is *gauche* to the amide group and *anti* to the ester group. The chirality was confirmed to be *S* from two-dimensional NMR spectroscopy. In the crystal, N—H⋯O and C—H⋯O hydrogen bonds and several short-contact inter­actions (2.07–3.45 Å) create chains parallel to [110]. The phenyl ring is disordered over two orientations in a 0.54 (2):0.46 (2) ratio.

## Related literature

The title compound is a precusor to novel chiral organocatalyts. For the synthesis, see: Naicker *et al.* (2011 ▶) and for related structures, see: Clegg & Elsegood (2003 ▶); Zalán *et al.* (2006 ▶)
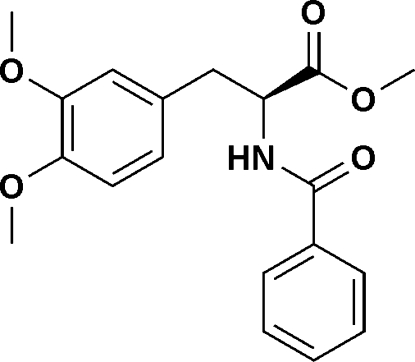


## Experimental

### 

#### Crystal data

C_19_H_21_NO_5_
*M* *_r_* = 343.37
Monoclinic, 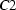

*a* = 20.331 (9) Å
*b* = 5.070 (3) Å
*c* = 17.580 (9) Å
β = 108.489 (8)°
*V* = 1718.5 (15) Å^3^
*Z* = 4
Mo *K*α radiation
μ = 0.10 mm^−1^
*T* = 173 K
0.75 × 0.05 × 0.03 mm

#### Data collection

Bruker Kappa DUO APEXII diffractometer
3865 measured reflections
1645 independent reflections
930 reflections with *I* > 2σ(*I*)
*R*_int_ = 0.077

#### Refinement

*R*[*F* ^2^ > 2σ(*F* ^2^)] = 0.077
*wR*(*F* ^2^) = 0.219
*S* = 0.99
1645 reflections
263 parameters
13 restraints
H-atom parameters constrained
Δρ_max_ = 0.33 e Å^−3^
Δρ_min_ = −0.35 e Å^−3^

### 

Data collection: *APEX2* (Bruker, 2006 ▶); cell refinement: *SAINT* (Bruker, 2006 ▶; data reduction: *SAINT*; program(s) used to solve structure: *SHELXS97* (Sheldrick, 2008 ▶); program(s) used to refine structure: *SHELXL97* (Sheldrick, 2008 ▶); molecular graphics: *OLEX2* (Dolomanov *et al.*, 2009 ▶); software used to prepare material for publication: *SHELXL97*.

## Supplementary Material

Crystal structure: contains datablock(s) I, global. DOI: 10.1107/S1600536811052767/hg5150sup1.cif

Structure factors: contains datablock(s) I. DOI: 10.1107/S1600536811052767/hg5150Isup2.hkl

Supplementary material file. DOI: 10.1107/S1600536811052767/hg5150Isup3.cml

Additional supplementary materials:  crystallographic information; 3D view; checkCIF report

## Figures and Tables

**Table 1:** Hydrogen-bond geometry (Å, °)

*D*—H⋯*A*	*D*—H	H⋯*A*	*D*⋯*A*	*D*—H⋯*A*
N1—H1⋯O5^i^	0.88	2.07	2.924 (9)	163
C2—H2⋯O5^i^	0.95	2.55	3.412 (11)	151

Symmetry code: (i) 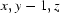.

**Fig. 1.**
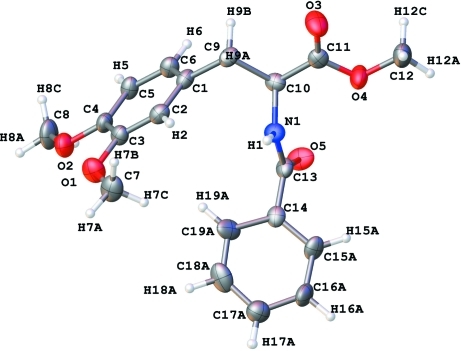
The molecular structure of the title compound with the atom numbering scheme. Displacement ellipsoids are drawn at the 40% probability level.

**Fig. 2.**
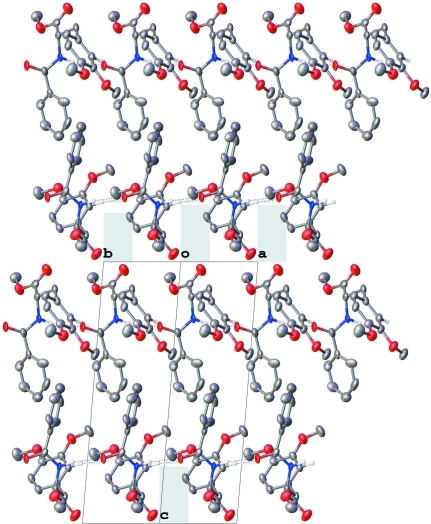
A partial projection of the title compound, viewed along the [110] plane.

## supplementary crystallographic information

### Comment

The title compound is a well known precusor to several biologically active compounds (Zalán *et al.*, 2006). In our laboratory it is being used as a precusor to novel chiral organocatalyts (Naicker *et al.* 2011).

There is only one analogous X-ray crystal structure that has a tertiary butyl group at the C12 position and a O—CH_2_-fluorenyl group is attached to the carbonyl carbon at C13 (Clegg & Elsegood, 2003). The title compound exists in a perfectly staggered conformation about the C9—C10 bond (Fig. 1). Similar to the analogous X-ray structure, the dimethoxyphenyl ring is *gauche* to the amide group and *anti* to the ester group.

The initial starting material for the synthesis of the title compound was optically pure *L*-DOPA, the chirality at the C8 atom remained unchanged during the synthesis and was confirmed to be *S* configuration from two-dimensional NMR spectroscopy.

The molecules in the crystal are connected by N1—H1···O5 (2.07 (9) Å) hydrogen bonds (Fig. 2), supported by a weak C2—H2···O5 (2.54 (11) Å) hydrogen bond from the dimethoxyphenyl ring which form chains parallel to the 110 plane (Table 2). In the analogous structure the same hydrogen bonds, have lengths of 2.36 Å and 2.42 Å respectively. In addition, there are several intermolecular short contact interactions (2.07–3.45 Å) within the crystal packing.

The phenyl ring is disordered with two orientations at 50% site occupancy.

### Experimental

Benzoic acid (0.5 g, 4.2 mmol) was dissolved was dissolved in DMF (15 ml) and THF (5 ml) followed by addition of HBTU (4.6 mmol), DIPEA (8.4 mmol) and (*S*)-methyl 2-amino-3-(3,4-dimethoxyphenyl)propanoate (1.0 g, 4.2 mmol). The reaction mixture was then stirred at room temperature until no more starting material could be detected by TLC analysis. The reaction mixture was poured into 30 volumes of chilled water; the mixture was then extracted thrice with ethyl acetate (20 ml). The combined extracts were dried over anhydrous sodium sulfate and then concentrated to dryness affording the crude product. This crude product was purified by column chromatography (50:50 EtOAc/Hexane, Rf = 0.6) to afford the product 1.30 g (92%) as a white solid. Melting point: 377–379 K.

Recrystallization from ethyl acetate at room temperature afforded crystals suitable for X-ray analysis.

### Refinement

All hydrogen atoms were positioned geometrically with C—H distances ranging from 0.95 Å to 1.00 Å and N—H distances 0.88Å and refined as riding on their parent atoms, with *U*_iso_ (H) = 1.2 - 1.5 *U*_eq_ (C or N). The final refinements were done with the Friedel pairs being merged. The phenyl ring is disordered with two orientations: the ring of C14, C15A, C16A, C17A, C18A and C19A and the other ring of C14, C15B, C16B, C17B, C18B and C19B, with C17A and C17B are at the common positions and the site occupancy factors were refined to 0.46 (2) and 0.54 (2) respectively. The bond distances of the disordered phenyl ring were restrained to 0.39 (1) Å. The hydrogen atom H1 (of N1) could not be located in the difference electron density maps and therefore was placed on a trigonal-planar position. This hydrogen position was justified by the presence of almost linear hydrogen bond N1—H1 to O5 of the neighbouring molecule.

### Crystal data

**Table:**

C_19_H_21_NO_5_	*F*(000) = 728
*M**_r_* = 343.37	*D*_x_ = 1.327 Mg m^−^^3^
Monoclinic, *C*2	Melting point: 378 K
Hall symbol: C 2y	Mo *K*α radiation, λ = 0.71073 Å
*a* = 20.331 (9) Å	Cell parameters from 3868 reflections
*b* = 5.070 (3) Å	θ = 2.1–25.2°
*c* = 17.580 (9) Å	µ = 0.10 mm^−^^1^
β = 108.489 (8)°	*T* = 173 K
*V* = 1718.5 (15) Å^3^	Needle, colourless
*Z* = 4	0.75 × 0.05 × 0.03 mm

### Data collection

**Table:**

Bruker Kappa DUO APEXII diffractometer	930 reflections with *I* > 2σ(*I*)
Radiation source: fine-focus sealed tube	*R*_int_ = 0.077
graphite	θ_max_ = 25.2°, θ_min_ = 2.1°
0.5° φ scans and ω scans	*h* = −24→23
3865 measured reflections	*k* = −5→5
1645 independent reflections	*l* = −20→21

### Refinement

**Table:**

Refinement on *F*^2^	Primary atom site location: structure-invariant direct methods
Least-squares matrix: full	Secondary atom site location: difference Fourier map
*R*[*F*^2^ > 2σ(*F*^2^)] = 0.077	Hydrogen site location: inferred from neighbouring sites
*wR*(*F*^2^) = 0.219	H-atom parameters constrained
*S* = 0.99	*w* = 1/[σ^2^(*F*_o_^2^) + (0.1349*P*)^2^] where *P* = (*F*_o_^2^ + 2*F*_c_^2^)/3
1645 reflections	(Δ/σ)_max_ = 0.001
263 parameters	Δρ_max_ = 0.33 e Å^−^^3^
13 restraints	Δρ_min_ = −0.35 e Å^−^^3^

### Special details

**Table:**

Experimental. Half sphere of data collected using the Bruker *SAINT* software package. Crystal to detector distance = 30 mm; combination of φ and ω scans of 0.5°, 120 s per °, 2 iterations.
Geometry. All e.s.d.'s (except the e.s.d. in the dihedral angle between two l.s. planes) are estimated using the full covariance matrix. The cell e.s.d.'s are taken into account individually in the estimation of e.s.d.'s in distances, angles and torsion angles; correlations between e.s.d.'s in cell parameters are only used when they are defined by crystal symmetry. An approximate (isotropic) treatment of cell e.s.d.'s is used for estimating e.s.d.'s involving l.s. planes.
Refinement. Refinement of *F*^2^ against ALL reflections. The weighted *R*-factor *wR* and goodness of fit *S* are based on *F*^2^, conventional *R*-factors *R* are based on *F*, with *F* set to zero for negative *F*^2^. The threshold expression of *F*^2^ > σ(*F*^2^) is used only for calculating *R*-factors(gt) *etc*. and is not relevant to the choice of reflections for refinement. *R*-factors based on *F*^2^ are statistically about twice as large as those based on *F*, and *R*- factors based on ALL data will be even larger.

### Fractional atomic coordinates and isotropic or equivalent isotropic displacement parameters (Å^2^)

**Table:**

	*x*	*y*	*z*	*U*_iso_*/*U*_eq_	Occ. (<1)
O1	0.4617 (3)	−0.3079 (14)	0.6767 (3)	0.0559 (17)	
O2	0.3895 (3)	0.0530 (15)	0.7181 (3)	0.0518 (16)	
O3	0.8139 (3)	−0.0862 (15)	0.9696 (4)	0.065 (2)	
O4	0.8358 (3)	0.2272 (15)	0.8951 (3)	0.0546 (18)	
O5	0.7020 (3)	0.5010 (12)	0.7518 (3)	0.0516 (17)	
N1	0.7096 (3)	0.0685 (14)	0.7830 (3)	0.0343 (15)	
H1	0.7116	−0.0934	0.7658	0.041*	
C1	0.5969 (4)	−0.0340 (16)	0.8513 (4)	0.0345 (19)	
C2	0.5642 (4)	−0.1938 (17)	0.7856 (4)	0.0372 (19)	
H2	0.5901	−0.3281	0.7705	0.045*	
C3	0.4958 (4)	−0.1618 (17)	0.7426 (5)	0.0368 (19)	
C4	0.4572 (4)	0.038 (2)	0.7634 (4)	0.0384 (19)	
C5	0.4888 (4)	0.197 (2)	0.8287 (5)	0.0392 (19)	
H5	0.4629	0.3301	0.8443	0.047*	
C6	0.5576 (4)	0.1620 (17)	0.8710 (5)	0.039 (2)	
H6	0.5789	0.2754	0.9149	0.047*	
C7	0.5016 (5)	−0.489 (2)	0.6473 (6)	0.058 (3)	
H7A	0.4712	−0.5801	0.6000	0.088*	
H7B	0.5234	−0.6184	0.6891	0.088*	
H7C	0.5376	−0.3927	0.6326	0.088*	
C8	0.3492 (4)	0.264 (2)	0.7364 (6)	0.064 (3)	
H8A	0.3016	0.2558	0.6997	0.097*	
H8B	0.3699	0.4343	0.7301	0.097*	
H8C	0.3489	0.2459	0.7918	0.097*	
C9	0.6713 (4)	−0.0682 (16)	0.8965 (4)	0.0359 (19)	
H9A	0.6840	−0.2550	0.8927	0.043*	
H9B	0.6786	−0.0289	0.9538	0.043*	
C10	0.7194 (4)	0.1063 (16)	0.8672 (4)	0.0345 (19)	
H10	0.7071	0.2937	0.8743	0.041*	
C11	0.7935 (4)	0.0700 (19)	0.9151 (4)	0.0374 (19)	
C12	0.9079 (4)	0.215 (3)	0.9399 (5)	0.059 (3)	
H12A	0.9334	0.3426	0.9182	0.089*	
H12B	0.9253	0.0368	0.9360	0.089*	
H12C	0.9143	0.2565	0.9962	0.089*	
C13	0.6978 (4)	0.2648 (17)	0.7294 (5)	0.0364 (19)	
C15A	0.7231 (10)	0.260 (5)	0.6026 (9)	0.044 (6)	0.46 (2)
H15A	0.7658	0.3476	0.6275	0.053*	0.46 (2)
C16A	0.7033 (8)	0.194 (5)	0.5219 (10)	0.052 (7)	0.46 (2)
H16A	0.7339	0.2343	0.4924	0.062*	0.46 (2)
C17A	0.6407 (4)	0.072 (2)	0.4826 (5)	0.054 (3)	0.46 (2)
H17A	0.6271	0.0291	0.4271	0.065*	0.46 (2)
C18A	0.5991 (10)	0.017 (5)	0.5305 (10)	0.060 (7)	0.46 (2)
H18A	0.5552	−0.0616	0.5050	0.072*	0.46 (2)
C19A	0.6163 (7)	0.067 (5)	0.6124 (9)	0.049 (6)	0.46 (2)
H19A	0.5872	0.0186	0.6430	0.059*	0.46 (2)
C14	0.6796 (4)	0.1945 (15)	0.6461 (4)	0.0349 (18)	
C15B	0.6839 (12)	0.376 (3)	0.5891 (8)	0.058 (6)	0.54 (2)
H15B	0.6999	0.5483	0.6066	0.070*	0.54 (2)
C16B	0.6663 (12)	0.321 (3)	0.5083 (9)	0.071 (8)	0.54 (2)
H16B	0.6716	0.4496	0.4715	0.085*	0.54 (2)
C17B	0.6407 (4)	0.072 (2)	0.4826 (5)	0.054 (3)	0.54
H17B	0.6283	0.0321	0.4270	0.065*	0.54 (2)
C18B	0.6327 (13)	−0.119 (4)	0.5342 (7)	0.070 (7)	0.54 (2)
H18B	0.6153	−0.2895	0.5163	0.084*	0.54 (2)
C19B	0.6516 (13)	−0.046 (3)	0.6141 (8)	0.058 (7)	0.54 (2)
H19B	0.6446	−0.1725	0.6506	0.070*	0.54 (2)

### Atomic displacement parameters (Å^2^)

**Table:**

	*U*^11^	*U*^22^	*U*^33^	*U*^12^	*U*^13^	*U*^23^
O1	0.059 (4)	0.044 (4)	0.062 (4)	−0.004 (3)	0.015 (3)	−0.017 (3)
O2	0.037 (3)	0.055 (4)	0.063 (3)	0.001 (3)	0.016 (3)	−0.004 (3)
O3	0.053 (4)	0.055 (5)	0.073 (4)	0.000 (3)	0.000 (3)	0.023 (4)
O4	0.040 (3)	0.057 (5)	0.065 (4)	−0.009 (3)	0.014 (3)	0.017 (4)
O5	0.090 (5)	0.011 (4)	0.058 (4)	−0.001 (3)	0.029 (3)	−0.002 (2)
N1	0.048 (4)	0.011 (3)	0.042 (3)	0.004 (3)	0.012 (3)	0.004 (3)
C1	0.037 (4)	0.026 (5)	0.047 (4)	0.005 (3)	0.022 (4)	0.006 (4)
C2	0.047 (5)	0.025 (5)	0.045 (5)	0.002 (4)	0.022 (4)	−0.002 (3)
C3	0.039 (5)	0.030 (5)	0.042 (5)	−0.006 (3)	0.015 (4)	−0.007 (4)
C4	0.030 (4)	0.046 (5)	0.044 (4)	−0.001 (4)	0.018 (4)	−0.002 (4)
C5	0.038 (4)	0.029 (5)	0.055 (5)	−0.002 (4)	0.022 (4)	−0.010 (4)
C6	0.042 (5)	0.033 (5)	0.046 (4)	−0.001 (4)	0.019 (4)	−0.009 (4)
C7	0.065 (6)	0.042 (7)	0.074 (6)	−0.011 (5)	0.030 (5)	−0.023 (5)
C8	0.034 (5)	0.069 (8)	0.096 (7)	0.002 (5)	0.029 (5)	−0.015 (6)
C9	0.049 (5)	0.019 (5)	0.045 (4)	−0.002 (3)	0.023 (4)	0.001 (3)
C10	0.047 (5)	0.018 (5)	0.037 (4)	0.002 (3)	0.010 (4)	0.000 (3)
C11	0.045 (5)	0.028 (5)	0.041 (5)	0.004 (4)	0.017 (4)	−0.009 (4)
C12	0.040 (5)	0.078 (8)	0.063 (5)	−0.010 (5)	0.021 (4)	−0.007 (6)
C13	0.036 (4)	0.026 (5)	0.047 (5)	−0.003 (4)	0.012 (4)	0.009 (4)
C15A	0.043 (11)	0.037 (14)	0.062 (13)	0.004 (11)	0.031 (10)	−0.009 (10)
C16A	0.032 (11)	0.070 (18)	0.060 (15)	0.004 (12)	0.024 (11)	0.010 (13)
C17A	0.055 (6)	0.056 (7)	0.053 (6)	0.007 (5)	0.018 (5)	−0.003 (5)
C18A	0.052 (14)	0.033 (16)	0.085 (17)	−0.001 (11)	0.009 (12)	−0.022 (12)
C19A	0.035 (11)	0.065 (16)	0.045 (12)	0.029 (13)	0.009 (9)	0.004 (11)
C14	0.038 (4)	0.017 (4)	0.050 (5)	0.004 (3)	0.014 (4)	0.006 (4)
C15B	0.075 (16)	0.033 (12)	0.072 (14)	−0.014 (12)	0.030 (11)	0.002 (10)
C16B	0.071 (16)	0.11 (2)	0.028 (10)	−0.030 (15)	0.014 (10)	−0.001 (11)
C17B	0.055 (6)	0.056 (7)	0.053 (6)	0.007 (5)	0.018 (5)	−0.003 (5)
C18B	0.110 (19)	0.051 (15)	0.039 (11)	−0.015 (15)	0.011 (11)	−0.004 (10)
C19B	0.077 (16)	0.047 (15)	0.043 (10)	−0.033 (12)	0.008 (10)	0.006 (9)

### Geometric parameters (Å, °)

**Table:**

O1—C3	1.365 (9)	C9—H9A	0.9900
O1—C7	1.426 (11)	C9—H9B	0.9900
O2—C4	1.355 (8)	C10—C11	1.484 (10)
O2—C8	1.445 (12)	C10—H10	1.0000
O3—C11	1.210 (10)	C12—H12A	0.9800
O4—C11	1.302 (10)	C12—H12B	0.9800
O4—C12	1.427 (9)	C12—H12C	0.9800
O5—C13	1.255 (11)	C13—C14	1.439 (10)
N1—C13	1.338 (10)	C15A—C14	1.380 (10)
N1—C10	1.442 (9)	C15A—C16A	1.387 (10)
N1—H1	0.8800	C15A—H15A	0.9500
C1—C6	1.386 (10)	C16A—C17A	1.386 (10)
C1—C2	1.395 (11)	C16A—H16A	0.9500
C1—C9	1.479 (10)	C17A—C18A	1.401 (10)
C2—C3	1.365 (10)	C17A—H17A	0.9500
C2—H2	0.9500	C18A—C19A	1.393 (10)
C3—C4	1.399 (11)	C18A—H18A	0.9500
C4—C5	1.382 (11)	C19A—C14	1.391 (10)
C5—C6	1.370 (10)	C19A—H19A	0.9500
C5—H5	0.9500	C14—C15B	1.383 (10)
C6—H6	0.9500	C14—C19B	1.385 (10)
C7—H7A	0.9800	C15B—C16B	1.378 (10)
C7—H7B	0.9800	C15B—H15B	0.9500
C7—H7C	0.9800	C16B—H16B	0.9500
C8—H8A	0.9800	C18B—C19B	1.384 (10)
C8—H8B	0.9800	C18B—H18B	0.9500
C8—H8C	0.9800	C19B—H19B	0.9500
C9—C10	1.526 (10)		
			
C3—O1—C7	117.6 (6)	C11—C10—H10	107.2
C4—O2—C8	116.9 (7)	C9—C10—H10	107.2
C11—O4—C12	118.4 (7)	O3—C11—O4	121.6 (7)
C13—N1—C10	123.9 (7)	O3—C11—C10	124.2 (8)
C13—N1—H1	118.0	O4—C11—C10	114.2 (7)
C10—N1—H1	118.0	O4—C12—H12A	109.5
C6—C1—C2	117.5 (7)	O4—C12—H12B	109.5
C6—C1—C9	121.5 (7)	H12A—C12—H12B	109.5
C2—C1—C9	121.0 (7)	O4—C12—H12C	109.5
C3—C2—C1	121.6 (7)	H12A—C12—H12C	109.5
C3—C2—H2	119.2	H12B—C12—H12C	109.5
C1—C2—H2	119.2	O5—C13—N1	120.7 (7)
O1—C3—C2	124.0 (7)	O5—C13—C14	121.7 (7)
O1—C3—C4	116.0 (7)	N1—C13—C14	117.6 (7)
C2—C3—C4	119.9 (7)	C14—C15A—C16A	118.6 (15)
O2—C4—C5	125.0 (8)	C14—C15A—H15A	120.7
O2—C4—C3	115.8 (7)	C16A—C15A—H15A	120.7
C5—C4—C3	119.1 (7)	C17A—C16A—C15A	122.7 (15)
C6—C5—C4	120.1 (8)	C17A—C16A—H16A	118.6
C6—C5—H5	120.0	C15A—C16A—H16A	118.6
C4—C5—H5	120.0	C16A—C17A—C18A	115.0 (12)
C5—C6—C1	121.8 (8)	C16A—C17A—H17A	122.5
C5—C6—H6	119.1	C18A—C17A—H17A	122.5
C1—C6—H6	119.1	C19A—C18A—C17A	125.7 (16)
O1—C7—H7A	109.5	C19A—C18A—H18A	117.2
O1—C7—H7B	109.5	C17A—C18A—H18A	117.2
H7A—C7—H7B	109.5	C14—C19A—C18A	114.9 (15)
O1—C7—H7C	109.5	C14—C19A—H19A	122.6
H7A—C7—H7C	109.5	C18A—C19A—H19A	122.6
H7B—C7—H7C	109.5	C15A—C14—C19B	103.8 (13)
O2—C8—H8A	109.5	C15B—C14—C19B	113.8 (11)
O2—C8—H8B	109.5	C15A—C14—C19A	123.0 (12)
H8A—C8—H8B	109.5	C15B—C14—C19A	105.2 (13)
O2—C8—H8C	109.5	C15A—C14—C13	120.1 (9)
H8A—C8—H8C	109.5	C15B—C14—C13	121.2 (9)
H8B—C8—H8C	109.5	C19B—C14—C13	124.8 (9)
C1—C9—C10	114.0 (6)	C19A—C14—C13	116.9 (9)
C1—C9—H9A	108.7	C16B—C15B—C14	123.8 (14)
C10—C9—H9A	108.7	C16B—C15B—H15B	118.1
C1—C9—H9B	108.7	C14—C15B—H15B	118.1
C10—C9—H9B	108.7	C15B—C16B—H16B	121.0
H9A—C9—H9B	107.6	C19B—C18B—H18B	122.3
N1—C10—C11	110.5 (6)	C18B—C19B—C14	126.4 (14)
N1—C10—C9	112.0 (6)	C18B—C19B—H19B	116.8
C11—C10—C9	112.3 (6)	C14—C19B—H19B	116.8
N1—C10—H10	107.2		
			
C6—C1—C2—C3	−0.8 (11)	C9—C10—C11—O4	−176.0 (7)
C9—C1—C2—C3	−179.0 (7)	C10—N1—C13—O5	7.9 (11)
C7—O1—C3—C2	−5.5 (12)	C10—N1—C13—C14	−171.6 (6)
C7—O1—C3—C4	171.7 (8)	C14—C15A—C16A—C17A	2(3)
C1—C2—C3—O1	178.2 (8)	C15A—C16A—C17A—C18A	−1(3)
C1—C2—C3—C4	1.1 (12)	C16A—C17A—C18A—C19A	−2(3)
C8—O2—C4—C5	4.8 (12)	C17A—C18A—C19A—C14	3(3)
C8—O2—C4—C3	−177.7 (8)	C16A—C15A—C14—C15B	−74 (2)
O1—C3—C4—O2	3.5 (11)	C16A—C15A—C14—C19B	36 (2)
C2—C3—C4—O2	−179.2 (7)	C16A—C15A—C14—C19A	0(3)
O1—C3—C4—C5	−178.9 (8)	C16A—C15A—C14—C13	−178.6 (17)
C2—C3—C4—C5	−1.5 (12)	C18A—C19A—C14—C15A	−2(3)
O2—C4—C5—C6	179.1 (8)	C18A—C19A—C14—C15B	39 (2)
C3—C4—C5—C6	1.7 (12)	C18A—C19A—C14—C19B	−71 (2)
C4—C5—C6—C1	−1.5 (12)	C18A—C19A—C14—C13	176.4 (16)
C2—C1—C6—C5	1.0 (11)	O5—C13—C14—C15A	65.0 (16)
C9—C1—C6—C5	179.2 (7)	N1—C13—C14—C15A	−115.6 (15)
C6—C1—C9—C10	−85.4 (9)	O5—C13—C14—C15B	17.4 (16)
C2—C1—C9—C10	92.6 (9)	N1—C13—C14—C15B	−163.1 (14)
C13—N1—C10—C11	−106.4 (8)	O5—C13—C14—C19B	−157.3 (16)
C13—N1—C10—C9	127.5 (8)	N1—C13—C14—C19B	22.2 (17)
C1—C9—C10—N1	−56.0 (9)	O5—C13—C14—C19A	−113.3 (14)
C1—C9—C10—C11	178.8 (7)	N1—C13—C14—C19A	66.2 (14)
C12—O4—C11—O3	−0.9 (13)	C15A—C14—C15B—C16B	80 (3)
C12—O4—C11—C10	177.4 (7)	C19B—C14—C15B—C16B	−4(3)
N1—C10—C11—O3	−123.6 (9)	C19A—C14—C15B—C16B	−44 (3)
C9—C10—C11—O3	2.3 (11)	C13—C14—C15B—C16B	−179.1 (18)
N1—C10—C11—O4	58.1 (9)	C13—C14—C19B—C18B	179.2 (19)

### Hydrogen-bond geometry (Å, °)

**Table:**

*D*—H···*A*	*D*—H	H···*A*	*D*···*A*	*D*—H···*A*
N1—H1···O5^i^	0.88	2.07	2.924 (9)	163
C2—H2···O5^i^	0.95	2.55	3.412 (11)	151

Symmetry codes: (i) *x*, *y*−1, *z*.
